# An Overview on Conventional and Non-Conventional Therapeutic Approaches for the Treatment of Candidiasis and Underlying Resistance Mechanisms in Clinical Strains

**DOI:** 10.3390/jof6010023

**Published:** 2020-02-10

**Authors:** Sara B. Salazar, Rita S. Simões, Nuno A. Pedro, Maria Joana Pinheiro, Maria Fernanda N. N. Carvalho, Nuno P. Mira

**Affiliations:** 1 Department of Bioengineering, Institute of Bioengineering and Biosciences, Instituto Superior Técnico, Universidade de Lisboa, 1049-001 Lisboa, Portugal; sara.salazar@ist.utl.pt (S.B.S.); ritasrmsimoes@gmail.com (R.S.S.); nunoalexandrepedro@tecnico.ulisboa.pt (N.A.P.); mariajoana@live.com.pt (M.J.P.); 2 Centro de Química Estrutural, Complexo I, Instituto Superior Técnico, Universidade de Lisboa, 1049-001 Lisboa, Portugal; fcarvalho@ist.utl.pt

**Keywords:** candidiasis, antifungal drugs, resistance to antifungals, non-conventional therapeutics, phytotherapeutics and probiotics, antimicrobials, *Candida*

## Abstract

Fungal infections and, in particular, those caused by species of the *Candida* genus, are growing at an alarming rate and have high associated rates of mortality and morbidity. These infections, generally referred as candidiasis, range from common superficial rushes caused by an overgrowth of the yeasts in mucosal surfaces to life-threatening disseminated mycoses. The success of currently used antifungal drugs to treat candidiasis is being endangered by the continuous emergence of resistant strains, specially among non-albicans *Candida* species. In this review article, the mechanisms of action of currently used antifungals, with emphasis on the mechanisms of resistance reported in clinical isolates, are reviewed. Novel approaches being taken to successfully inhibit growth of pathogenic *Candida* species, in particular those based on the exploration of natural or synthetic chemicals or on the activity of live probiotics, are also reviewed. It is expected that these novel approaches, either used alone or in combination with traditional antifungals, may contribute to foster the identification of novel anti-*Candida* therapies.

## 1. Relevance of Candidiasis within the Spectrum of Fungal Infections

In recent years, the number of fungal infections has risen significantly, being today estimated to affect, yearly, around 150 million people and cause 1.5 million deaths [1,2]. These infections range from superficial rushes in the mucosas, in the skin or in the nails, to systemic infections, in which the fungal cells disseminate in the bloodstream and may end up colonizing any major internal organ [1]. *Candida* species are among the more relevant etiological agents causative of superficial and invasive fungal infections. Vulvovaginal candidiasis (the common name attributed to infections caused by *Candida* spp. in the vaginal tract) is estimated to affect 70% to 75% of women worldwide, 5% to 8% of these in a recurrent manner [3]. The incidence of invasive candidiasis annually is estimated to be 700,000 infections, with associated mortality rates close to 50%, especially in countries where no adequate antifungal therapy is available [1,2]. Different from other relevant fungal pathogens, such as those belonging to the *Aspergillus* or *Cryptococcus* genera, *Candida* spp. are part of the human commensal microbiota colonizing the skin, the genitourinary or the gastrointestinal tracts [4]. Under certain conditions, such as reduced activity of the host immune system, prolonged use of antibiotics or chemotherapy, these commensal populations can overgrow, triggering more serious (in some cases life-threatening) infections [2]. *C. albicans* is the more relevant species in causing superficial and invasive candidiasis, but a growing incidence of non-albicans *Candida* species (usually known as NACS) is reported [5]. *C. glabrata*, *C. tropicalis*, *C. parapsilosis* and *C. krusei* are among the more relevant NACS, accounting, together with *C. albicans, for* more than 80% of all described cases of candidiasis [6,7]. The crude mortality rate associated to infections caused by NACS has been reported to exceed those attributed to *C. albicans* (ca. 37%) reaching in the highest cases ~50% for *C. glabrata* and ~59% for *C. krusei* [8]. This epidemiological shift from *C. albicans* to NACS is believed to result from a selective pressure caused by the massive utilization of azoles in prophylactic and active treatments that resulted in the selection of species innately more tolerant to these drugs. The use of better diagnosis methods to identify isolates in the clinical setting is another relevant factor as in the past the identification of the *Candida* isolates may have not been as accurate as it is today [9].

## 2. Available Antifungals against *Candida* spp. and Their Modes of Action

**Figure 1:**
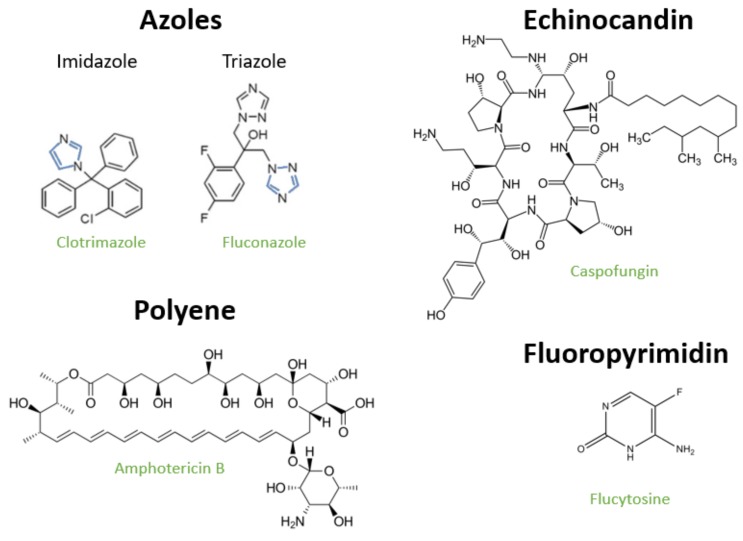
Representative examples of the antifungals currently available to treat candidiasis. Chemical structure of representative examples of antifungals (azoles, echinocandins, polyenes and fluoropyrimidines) available, with the class of the drug being highlighted in black bold while the name of the drugs is shown in green. The nitrogen-based ring that distinguishes imidazoles (clotrimazole) from triazoles (fluconazole) is highlighted in blue.

The development of antifungal drugs is limited by the similarity between fungal and human cells, making it therefore difficult to identify molecules that specifically target the microbial cell while not damaging the host. The classes of antifungals available include azoles, polyenes and echinocandins. These target the biosynthesis of ergosterol or the cell wall, two cellular traits absent in mammalian cells (Figure 1). 5-fluorocytosine, a fluoropyrimidine, is also used to treat candidiasis but in this case the mechanism is more general as it targets DNA synthesis (Figure 1). A small description of the mechanisms of action of these molecules and the underlying resistance mechanisms is provided in the following subsections.

### 2.1. Polyenes

**Figure 2:**
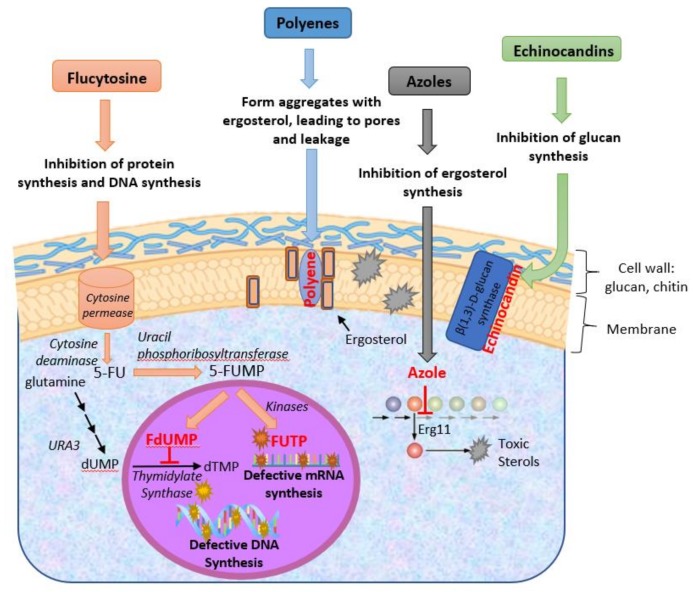
Schematic representation of the known mechanisms of action of the different classes of antifungals available for treatment of candidiasis. 5-FU—5-fluorouracil; 5-FUMP—5-fluorouridine monophosphate; FdUMP—5-fluorodeoxyuridine monophosphate; FUTP—5-fluorouridine triphosphate.

The better studied and more largely used polyene is amphotericin B which was the first antifungal developed for the treatment of disseminated candidiasis [10]. Nystatin is also used against *Candida* although only to treat oral infections [11]. The high lipophilicity of polyenes renders them able to penetrate the phospholipid bilayer of the plasma membrane where they bind ergosterol and promote the formation of pores (Figure 2) [12]. Necessarily, this perturbs the action of the plasma membrane as a selective barrier and a matrix for proteins. Despite its potent effect against *Candida*, the usefulness of amphotericin B is limited by its nephrotoxicity [13]. Although safer formulations to vehicle this drug have been developed (mostly based on the use of liposomes), its high cost remains an impediment and it is mostly used as a second-line therapy [13]. All *Candida* species show susceptibility to polyenes but in the case of *C. glabrata* and *C. krusei* the use of maximal doses is recommended (Table 1) [13].

### 2.2. Azoles

Azoles comprise the largest family of antifungals used against *Candida*. The first azoles used in clinical practice were clotrimazole and miconazole that were approved for use in 1969, followed by ketaconazole in 1981 [14]. These three drugs are all imidazoles since they harbour an imidazole ring in their structure (as shown in Figure 1). The usefulness of clotrimazole and miconazole as antifungals was limited by their inhibitory effect on the human hepatic CYP enzymes [15]. As a response to that, in the early 90s the triazoles fluconazole and itraconazole were introduced in the market, showing improved pharmacokinetic profile, a broader spectrum of antifungal activity and a lower inhibitory effect against the human CYP450 system [14]. In the early 2000s voriconazole emerged, being advantageous by showing higher activity against the more azole-resilient NCAS species, compared to fluconazole or itraconazole [14]. Today imidazoles are mostly used for the treatment of superficial candidiasis, while triazoles are preferred for the treatment of invasive candidiasis [14,16]. Regardless of the family they belong to, azoles act by inhibiting the activity of the lanosterol-14α-demethylase enzyme (encoded by the *ERG11* gene) that is involved in ergosterol biosynthesis. As a result of this inhibition, azole-exposed fungal cells accumulate toxic sterols in the plasma membrane dramatically affecting its permeability (Figure 2) [12]. *C. glabrata* and *C. krusei* show less susceptibility to azoles than the remaining *Candida* spp. and higher doses are recommended to treat infections caused by these species (Table 1).

### 2.3. Fluoropyrimidines

The fluoropyrimidine more commonly used in the treatment of candidiasis is 5-flucytosine (5-FC), which enters fungal cells through cytosine transporter(s) being afterwards metabolized via the pyrimidine salvage pathway to 5-fluorouracil (5-FU), considered the active form of 5-FC (Figure 2). 5-FU incorporates in RNA, causing premature chain termination, and inhibits the activity of thymidylate synthase, an enzyme essential for DNA synthesis (Figure 2) [12,17]. With the exception of *C. krusei*, the remaining *Candida* spp. are susceptible to 5-FC (Table 1). Although the enzymes that drive conversion of 5-FC into 5-FU are not present in mammalian cells [12], bacteria living in the human gut were shown to efficiently convert 5-FC into 5-FU [17] thereby explaining the toxic effects reported in patients under 5-FC therapy. Due to its toxic effects, 5-FC is given to patients in low concentration and in combination with other antifungals [13].

### 2.4. Echinocandins

Echinocandins are the only new class of antifungals discovered in recent years [1]. These are commercially available in three forms: caspofungin, anidulafungin and micafungin. Two more recent molecules, rezafungin and biafungin, have been recently described but its use in the clinical setting is not yet established as their efficacy is still under assessment in clinical trials. Compared to the already available echinocandins, rezafungin and biafungin show higher activity, lower toxicity and fewer drug interactions [18,19,20]. Echinocandins act by inhibiting the catalytic subunits of β-(1,3)-d-glucan synthase, essential for cell wall synthesis. Consequently, no elongation of (1,3)-β-d-glucans is observed in fungal cells exposed to echinocandins, rendering them highly susceptible to lysis (Figure 2) [12]. Echinocandins show efficacy against all *Candida* species, although *C. parapsilosis* has been found to be intrinsically less susceptible [13]. Due to their safety profile and fungicidal activity, echinocandins are frequently used as the primary treatment of invasive candidiasis [13].

## 3. Incidence of Antifungal Resistance and Underlying Mechanisms

In recent years, the number of resistant strains among *Candida* increased prominently, especially among NACS [23,24,25] (Table 2). Among the different antifungal classes, the highest percentage of resistance is observed for azoles, as detailed in Table 2. It is believed that this growing emergence of resistance to azoles is linked to the massive use of fluconazole in prophylaxis of patients considered at risk of suffering an infection caused by *Candida* [26,27,28,29]. The use of agricultural fungicides structurally similar to clinical azoles exerted another layer of pressure for the selection of more azole-tolerant *Candida* strains [30,31]. Although resistance to echinocandins and amphotericin B is very low, a slight, but detectable, increase in the emergence of resistant strains has been observed for *C. glabrata* and *C. krusei* [32] (Table 2).

### 3.1. Molecular Mechanisms Underlying Resistance to Antifungals in Clinical Strains

In this section the main mechanisms behind resistance of clinical *Candida* strains to the different classes of antifungals will be described. In general, these mechanisms of resistance can be summarized as involving the evolution of adaptive responses aiming to counteract the deleterious effects of the antifungal (e.g., reducing drug efficacy by changing the target) or to reduce the internal concentration of the drug (e.g., through the overexpressing drug-efflux pumps). The mechanisms already characterized as underlying resistance to to azoles in clinical isolates were gathered in Table 3, while those conferring tolerance to echinocandins, polyenes or 5-FU in clinical strains are detailed in Table 4.

#### 3.1.1. Azoles

**Figure 3:**
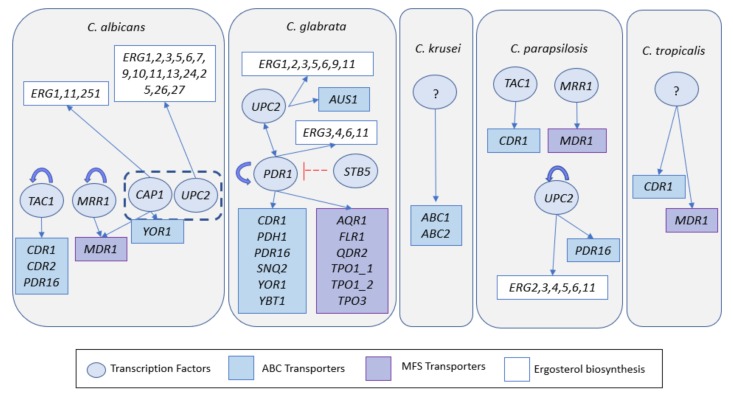
Schematic representation of regulatory associations between regulators involved in azole resistance and genes encoding multidrug resistance efflux pumps demonstrated to be involved in azole resistance in *Candida* spp. The information concerning the regulatory associations between transcription factor and target genes was retrieved from the PathoYeastract database [84]. Although ABC-MDR and MFS-MDR transporters involved in azole resistance in *C. krusei* and *C. tropicalis* had been identified, until so far the regulators of these genes remain to be characterized.

Resistance to azoles in *Candida* has been largely associated to modifications or overexpression of the drug target Erg11, modifications in the ergosterol pathway or overexpression of genes encoding drug-efflux pumps (Table 3). Numerous single nucleotide polymorphisms (SNPs) were reported to occur in the azole-target enzyme Erg11 encoded by *C. albicans, C. krusei* or *C. tropicalis*, it being thought that these mutations reduce the inhibitory effect of the azole over the enzyme [40,41,42,43,44]. Overexpression of *ERG11* has also been described as a mechanism driving resistance in *C. albicans*, *C. parapsilosis* and *C. tropicalis* isolates [41,42,45,46]. The higher transcription of *ERG11* in these azole-resistant isolates has been shown to result from these strains upregulating or encoding hyperactive forms of the Upc2 transcription factor, a strong positive regulator of *ERG11* gene [47,48,49] (Figure 3). Differently, the *CgERG11* allele encoded by *C. glabrata* azole-resistant isolates is, in the vast majority of the cases, identical to the one encoded by susceptible strains [50,51,52,53]. No link between the overexpression of *CgERG11* and increased resistance to azoles could also be established in *C. glabrata* [51,53,54] suggesting that this species has evolved responses to handle azole stress distinct from those verified in *C. albicans* or *C. parapsilosis*.

The induction of the activity of drug-efflux pumps has been observed in several azole-resistant isolates belonging to the different *Candida* species [42,46,49,55,56,57,58,59,60,61,62,63,64,65,66,67,68,69,70]. The more studied drug efflux pumps linked to azole resistance are those belonging to the ATP-binding cassette (ABC) superfamily which include in *C. albicans* CaMdr1, CaCdr1 and CaCdr2 [55,56,71]; in *C. glabrata*, CgCdr1, CgCdr2 and CgPdh1 [59,60,61]; in *C. krusei*, CkAbc1 and CkAbc2 [69]; in *C. parapsilosis* CpCdr1 and in *C. tropicalis* CtCdr1 [42,46]. More recently, multi drug resistance (MDR) transporters belonging to the Major Facilitator Superfamily (MFS) have also been implicated in tolerance of different *Candida* species to azoles including CaMdr1 in *C. albicans*, *C. parapsilosis* and *C. tropicalis* [42,46,49] and CgTpo1_1, CgTpo3 and CgQdr2 in *C. glabrata* [72]. Although the influence of these transporters in mediating resistance in clinical isolates has not been studied at the same extent as those of the ABC superfamily, promising results had been obtained in a recent study showing a positive correlation between the expression of the *C. glabrata* CgAqr1, CgTpo1_1, CgTpo3 and CgQdr2 MFS-MDR transporters and resistance to clotrimazole [70]. In this study it was also shown that the deletion of *CgTPO3* abolishes resistance to clotrimazole in one of the identified resistant clinical isolates [70]. The model that is generally accepted to explain the positive effect of the ABC and MFS transporters in drug resistance is their role in directly mediating the extrusion of the drugs, however, from the biochemical point of view this model is difficult to accept considering the wide structural divergence of the hypothesized substrates, [73]. Indeed, more recent studies performed in the eukaryotic model yeast *S. cerevisiae* show that ABC and MFS-MDR transporters have physiological substrates whose transport may affect the partition of the drugs between the intra- and the extracellular environment, [73]. Specifically, some MDR transporters have been shown to influence the lipid composition of the plasma membrane, by promoting the transport of phospholipids and/or ergosterol, which thereby may affect the diffusion rate of the drugs across the membrane, [74]. It was recently shown that deletion of the poorly characterized *C. albicans* ABC transporter CaRoa1 results in increased membrane rigidity and, consequently, in a reduced intracellular concentration of azoles [75]. Further studies in *Candida* spp. are required to clarify whether the observed positive effect of ABC and MFS- MDR transporters in reducing internal concentration of azoles is exerted directly or indirectly, via the transport of another physiological substrate and the relevance of these mechanisms in driving resistance in clinical isolates.

In all cases described so far, the higher activity of MDR pumps is linked to their higher expression in the azole-resistant isolates [72]. In *C. albicans* and in *C. glabrata* the transcriptional regulation of these drug-efflux pumps is under a tight control of the pleiotropic drug resistance network (or PDR) that in *C. glabrata* is dependent of the CgPdr1 regulator [76] while in *C. albicans* is controlled by CaTac1 [77] (Table 3). Further studies, exploring gene-by-gene or genome-wide approaches, have implicated other regulators in the transcriptional regulation of drug-efflux pumps or ergosterol metabolism under azole stress including CaMrr1 and CaCap1 in *C. albicans* [78,79]; CgStb5 in *C. glabrata* [80], Upc2 in *C. albicans*, *C. tropicalis, C. parapsilosis* and *C. glabrata* [48,49,57,81] and CpTac1 and CpMmr1 in *C. parapsilosis* [41,82]. The knowledge gathered on the regulatory associations between known transcription factors involved in azole resistance and MDR pumps is briefly summarized in Figure 3. In the case of the less-studied species *C. tropicalis* and *C. krusei,* the regulators of the identified drug-efflux pump-encoding genes are not yet identified. Nonetheless, similarity searches revealed that these species encode proteins showing similarity to CaTac1 (*CTRG_05307* in *C. tropicalis*) and to CaMrr1 (CTRG_02269 in *C. tropicalis* and JL09_3889 in *C. krusei*) [83].

The overexpression of drug-efflux pump-encoding genes results, in many cases, from the occurrence of activating mutations in the coding sequence of the corresponding regulators [41,45,78,82,85,86,87,88,89]. This type of mechanism has been documented for CgPdr1 in *C. glabrata*; for CaTac1, CaMrr1 and CaUpc2 in *C. albicans* and for CpMrr1 and CpTac1 in *C. parapsilosis* [41,45,78,82,85,88]. An important feature of these “hyper-active” alleles is that they become active even when azoles are absent [78,85,88]. Interestingly, it was recently shown that *C. albicans* strains harboring CaTac1 gain-of-function alleles exhibit a decreased fitness in vivo, specially when challenged with stresses other than azoles [90]. It thus seems that specialization of the cells to improve azole stress at the expense of CaTac1 hyper-activation results in reduced capacity to handle unrelated stresses. In the same line, the expression of CgPdr1 gain-of-function alleles were also hypothesized to be linked with a reduced tolerance of *C. glabrata* to organic acids [51,68,89,91].

#### 3.1.2. Flucytosine, Echinocandines and Polyenes

Acquired resistance to polyenes in clinical isolates is rare and the few studies correlate that phenotype with a reduction of ergosterol content in the plasma membrane of the resistant isolates [50,92,93,94,95,96]. These events are generally associated with the occurrence of SNPs that inactivate genes of the ergosterol biosynthetic pathway and thereby alter the sterol content of the membrane, this being described in *C. albicans, C. glabrata* and *C. tropicalis* (detailed in Table 4) [50,92,93,94,95,96]. No significant link between the activity of drug-efflux pumps and resistance to polyenes has been identified in resistant isolates belonging to the different *Candida* species. Although amphotericin B-resistant isolates had been identified in *C. krusei* and *C. parapsilosis* [97,98], the underlying mechanisms remain to be disclosed (Table 2). The echinocandin-resistance phenotype exhibited by the small number of identified resistant *Candida* isolates was attributed to mutations in the β-1,3-glucanase-encoding genes *FKS1* and *FKS2* genes [99,100,101,102,103]. These mutations are thought to reduce the sensitivity of the proteins to the drug [100] (Table 4) [99,100,101,102,103]. The naturally high tolerance to echinocandins of *C. parapsilosis* as well as of the closely related species *C. orthopsilosis and C. metapsilosis,* was also suggested to result from these species encoding a *CpFKS1* allele less sensitive to echinocandins [104]. Up to now, increased activity of drug-efflux pumps has not been identified as a relevant mechanism by which clinical isolates acquire resistance to echinocandins. Concerning flucytosine, resistance in clinical isolates has been linked to modifications on the coding sequence of the Fcy cytosine permease or in the uracil phosphotransferase Fur1 (Table 4 and Figure 1) [105,106,107,108,109,110]. Resistance of some *C. tropicalis* isolates was linked to the emergence of mutations in *CtURA3* gene, encoding the enzyme involved in the metabolization of UMP, the natural substrate of thymidylate synthase (Figure 1) [108,109]. It is thought that this mutation increases the synthesis of UMP compensating for the loss of this metabolite that will occur with formation of 5-FdUMP (Figure 1) [105].

### 3.2. Antifungal Resistance Driven by Large-Scale Genomic Alterations

A recent genomic analysis has unveiled an important role for the inactivation of the *CgMSH2* gene as a driver of resistance to azoles, echinocandins and amphotericin B in *C. glabrata* while colonizing the host [129]. The *CgMSH2* gene encodes a protein involved in DNA repair and its inactivation (promoted by frameshift mutations in the coding sequence) leads to increased genetic diversity in the *C. glabrata* population. As such, isolates harbouring inactive *CgMSH2* alleles rapidly acquired resistance to azoles, echinocandins or amphotericin B resulting from the rapid acquisition of beneficial mutations in CgPdr1, in CgFks1 or CgFks2 or in CgErg6 [129]. After this pioneering work, several epidemiological studies have focused their attention on the prevalence of resistant strains harbouring inactivated *CgMSH2* alleles, the percentages observed ranging between 5% and 17% [129,130,131,132]. Around 50% of the susceptible isolates examined in these studies were also found to harbour inactivated *CgMSH2* alleles [129,130,131,132], suggesting that this mechanism does not per se assure antifungal resistance. Deletion of the *C. albicans CaMSH2* gene was also found to result in drug resistance [133], however, up to now this mechanism has not been described to underlie the resistance phenotype in clinical isolates. The genomic plasticity exhibited by *C. albicans* and *C. glabrata* has also been found to contribute to increased drug resistance in these species. In specific, in azole-resistant clinical *C. glabrata* isolates it has been described the duplication of chromosomes that include the *CgCDR1*, *CgPDH1* or *CgERG11* genes, as well as the formation of mini-chromosomes harbouring several copies of genes encoding *CgCDR1* or *CgPDH1* [124,134]. The diploid nature of *C. albicans* has also been found to underlie the appearance of hyperactive alleles of genes involved in azole-resistance (e.g., *CaERG11* and *CaTAC1*) [120,135,136,137]. More recently, mis-translation of serine tRNAs in leucine at CUG codons, a well known specific trait of *C. albicans*, has also been linked with an accelerated resistance rate to fluconazole [137,138] while loss of heterozygosity was reported to underlie resistance to flucytosine in *C. tropicalis* [139].

## 4. Novel Approaches for the Development of Anti-*Candida* Agents

The persistent increase in the emergence of strains resistant to currently used antifungals has been paving the way for the development of new approaches that can be used to prevent growth of *Candida* spp. and that can be further considered as interesting alternatives as new anti-Candida therapies. The main results obtained in these different approaches are described in the following sections, together with a discussion on what are the current challenges or limitations in knowledge that still persist.

### 4.1. Phytotherapeutics

Systematic testing of compounds from natural sources including substances/extracts produced by animals, plants or microorganisms, have resulted in the identification of many molecules that inhibit growth of *Candida* cells. When produced by plants these substances are named phytotherapeutics, these being attractive since they are naturally perceived by consumers as less toxic and safer than common pharmaceuticals [140]. Currently, there is an increasing number of phytotherapeutics being identified as efficient against *Candida* species including extracts isolated from garlic (*Allium sativum* L., *Tulbaghia alliace* or *Tulbaghia violacea*), coconut (*Cocos nucifera*) or virgin coconut oil, mint (*Mentha piperita* L.) or sage (*Salvia officinalis* L.) [140,141]. A frequent limitation of this type of approaches is the difficulty in isolating the molecules responsible for the observed inhibitory effect over *Candida* since frequently these natural extracts are complex and used without further processing.

### 4.2. Redesign of “Old Antifungals”

The “redesign” of common antifungals is also an approach that has been explored to obtain molecules with inhibitory potential against *Candida*. The most paradigmatic examples are the new formulations of amphotericin B, which include lipid-associated and liposomal formulations showing higher fungal targeting and reduced toxicity against the host [142]. Within the same line azole-like molecules have also been obtained showing increased antifungal potency against all *Candida* species, compared to the efficacy exhibited by fluconazole [143]. These modified azoles, named ATTAF-1 and ATTAF-2, share general structural features with triazole alcohols, however, their mode of action appears to differ from the one of fluconazole which is an important trait to sensitize resistant strains [143]. Further on, Shrestha et al. (2017) developed a series of 27 alkylated variants of fluconazole, some of which presented a low hemolytic activity, low cytotoxicity and strong inhibitory potential against several *Candida* species [144]. Although the range of minimal inhibitory concentrations obtained was fairly wide, these compounds proved efficient against both *C. albicans* and non-*C. albicans* species and were observed to target the ergosterol biosynthetic pathway by inhibiting the sterol 14a-demethylase enzyme instead of targeting the *ERG11* gene [144].

### 4.3. New Compounds Obtained by Chemical Synthesis

The synthesis of entirely new compounds obtained by chemical synthesis, either or not involving metallic elements, has also been largely explored to obtain compounds with anti-*Candida* potential. Table 5 provides a systematic overview on a large cohort these “new chemicals”. Many of those new chemicals have silver in their structure, which is interesting since silver has been used since the times of ancient Greece as an antimicrobial. Examples of the Ag-containing compounds synthesized include those containing camphorimines, tetraazoles, albendazoles or phenantrolines as ligands (Table 5) [145,146,147,148]. Complexes with other metal centers like copper, cobalt, nickel or iron; or even with metals not usually used as bioagents, such as tin, chromium, cadmium or lead [149,150,151,152,153,154,155,156,157], have also been synthesized and shown to display moderate anti *C. albicans* activity (Table 5). Polinuclear complexes (based on Cu, Cd or Ni), particularly those harbouring ferrocenyl derived ligands, were also reported to have high activity against *C. albicans* (Table 5) [158]. More recently the use of a Ru(III) perylene complex has also been reported to be interesting as anti-*Candida* agent through photodynamic inactivation [159]. Although some of these complexes revealed a marked potential to constitute novel anti-*Candida* agents, their mechanism of action remains elusive in most cases, being also necessary to investigate their ability to inhibit growth of strains that are resistant to currently used antifungals. Another aspect of relevance is the fact that in the majority of the studies performed the compounds were not tested against NACS or against clinical strains that are, in general, more difficult to inhibit than laboratory strains.

### 4.4. Nanoparticles

Considering the recent interest in the use of nanoscale materials as antimicrobial agents, due to their high surface area to volume ratio that gives them unique chemical and physical properties [160], a number of studies have focused on the development and exploration of silver nanoparticles (AgNPs) as anti-*Candida* agents [161,162,163]. In these studies, silver nanoparticles are synthesized using organic or inorganic reductive agents (e.g., silver nitrate or citrate) [161,162] which promote the formation of metallic silver (Ag^0^), followed by agglomeration into oligomeric clusters that eventually result in the formation of metallic colloidal silver particles [164]. The exact mechanism by which AgNPs exert toxicity against *Candida* spp. remains a bit elusive, although evidence has been obtained suggesting that they may perturb the cellular envelope causing a disruption of the plasma membrane potential and consequent damage and leakage of cell constituents [163]. Concerning this matter, an interesting result was obtained with camphorimine-based complexes, being demonstrated that *C. albicans*, but not *C. parapsilosis*, *C. tropicalis* or *C. glabrata*, were able to mediate the conversion of Ag(I) into AgNPs [145].

### 4.5. Use of Probiotics and Antimicrobial Peptides

For a long time, it has been known that the use of probiotics can be beneficial for the treatment of mucosal candidiasis, specially, for vaginal candidiasis. In this sense, a few products are currently available in the market mostly based on the use of lactobacilli, considering the well-known track record of these species as probiotics [165]. A few examples of these products are described in Table 6. Probiotics are defined as “live microorganisms that, when administered in adequate amounts, provide a health benefit on the host” [166]. In the vaginal tract, the microbiota is largely dominated by lactobacilli, with *L. gasseri*, *L. jensenii* and *L. crispatus* being among those most abundant [167,168]. A decreased abundance in these microbial species appears to correlate with increased activity of pathogens, including of *C. albicans* and *C. glabrata* [169,170]. These results support the long-standing use of probiotics in the treatment of vaginal candidiasis. The mechanisms by which these lactobacilli species inhibit growth of pathogens, and of *Candida* in particular, remains to be elucidated, as well as the genes that mediate this interaction. Nonetheless, the evidence gathered so far (obtained using lactobacilli species differing from those that are indigenous of the vaginal tract) suggest that production of lactic-acid-concomitant acidification of the vaginal pH is on the basis of the protective effect of lactobacilii against vaginal pathogens [171]. Although this can be hypothesized for bacteria that are generally sensitive to low pH, this is not the case of yeasts that grow very well under acidic pHs. Indeed, a recent study performed with the supernatant of vaginal lactobacilli species (*L. crispatus*, *L. gasseri* and *L. vaginalis*) showed no significant correlation between the amount of lactic acid present and the inhibition of *Candida* [172] and concentrations of lactic acid similar to those found to be present in the vaginal tract (even under conditions of eubiosis) were also found not to significantly affect growth of *C. albicans* or *C. glabrata* [173,174]. It thus remains to be established what is the contribution that lactic acid production may have in the inhibition of *Candida* and of other vaginal pathogens. Other mechanisms by which vaginal lactobacilli are hypothesized to control the activity of *Candida* species in the vaginal tract is by competing for adhesion sites in the epithelial cells, by secreting biosurfactants that may decrease fungal binding to host surfaces and by secreting to the environment hydrogen peroxide and bacteriocins [171]. Interestingly, it was recently shown that invasive candidiasis from the gut can be restrained by commensal bacteria [175] which opens the door to the development of probiotics not only for the treatment of vaginal candidiasis but also for patients that may be at a high risk of developing systemic candidiasis caused by commensal *Candida* populations found in the gut such as those subjected to massive invasive surgeries.

## 5. Conclusions

Although extensive knowledge about the molecular mechanisms by which *Candida* spp. surpass the deleterious effects of antifungals has been collected, the translation of that knowledge to the understanding of which of these mechanisms play a role in the stressful environment of the host is still limited. In this review, we aimed at providing that picture, focusing what is actually described to mediate resistance in clinical isolates. The modification of the drug target and the overexpression of genes playing a detrimental role in antifungal tolerance determined by the adjustment of regulatory circuits (through modification of pivotal regulators in drug resistance such as CaTac1 or CgPdr1) and/or the occurrence of chromosomal rearrangements, comprise the vast majority of what is known to mediate antifungal tolerance in resistant isolates. However, there is still a road to pursue in this since the resistance of several resistant isolates cannot be explained by these mechanisms strongly suggesting that other antifungal-resistance genes remain to be identified. It is possible that the difficulty in mimicking in the laboratory the stressful environment of the host complicates the identification of these genes and, in this field, it is expected that extensive genomic analyses of resistant isolates may help to shed light on this. The full clarification of this panoply of resistance genes and mechanisms is essential not only to improve the success of treatments and improve the outcomes of candidiasis, but also to develop more efficient diagnosis tools that could rapidly provide clinicians a fast response on how to fine-tune treatments. It also seems clear that the development of non-conventional therapies, focused on biological targets other than those that are targeted by already used antifungals, is essential considering the persistent increase in the emergence of strains resistant to azoles and, less significantly, to echinocandins. Although much has been done in this field and promising results had been obtained, especially in the identification of new chemicals showing a robust anti-*Candida* effect, it remains to be established in many cases if indeed these compounds are able to sensitize antifungal-resistant isolates, and what their spectrum of activity against NACS is. In almost all cases it is also lacking the characterization of the toxicological effects of these drugs/compounds/probiotics in mammalian cells as well as their pharmacokinetic profile. Further investigation in this field is therefore essential to assure that alternative antifungals will be provide to the community in the mid-term.

## Figures and Tables

**Table 1:** General susceptibility patterns of *Candida* species to antifungal drugs used in the treatment of candidiasis (adapted from [21,22]). S—susceptible; S-DD—susceptible dose-dependent; I—intermediate; R—resistant.

Species	Imidazoles	Triazoles	Flucytosine	Ampho. B	Echinocandins
*C. albicans*	S to R	S	S	S	S
*C. tropicalis*	S	S	S	S	S
*C. parapsilosis*	S	S	S	S	S to I
*C. glabrata*	S-DD to R	S-DD to R	S	S to I	S
*C. krusei*	S to R	S-DD to R	I to R	S to I	S

**Table 2:** Percentage of isolates among the five more prevalent *Candida* spp. exhibiting resistance to azoles, echinocandins, flucytosine or amphotericin B (amphoB), as reported by surveillance epidemiological studies [22,32,33,34,35,36,37,38,39]. ** The high range of percentages found for *C. krusei* results from this species showing highly divergent susceptibilities to different imidazoles or triazoles (e.g., most strains are largely resistant to fluconazole but susceptible to voriconazole).

Species	Imidazoles	Triazoles	Echinocandins	Flucytosine	Ampho. B
*C. albicans*	0–54	0–16.6	0	0.3–4.3	0
*C. glabrata*	0–50.5	6.9–15.7	1.1–1.5	0–0.6	0–1.6
*C. tropicalis*	4–14	4.1–6.1	0	1–12.5	0–1
*C. parapsilosis*	0–2	1.8–14	0	0–1.4	0
*C. krusei*	0–73.1 **	2.8–100 **	0–2.8	1–16	0–12

**Table 3:** Summary of the mechanisms of resistance registered in azole-resistant *Candida* isolates, as described in [40,41,42,43,45,46,47,48,49,50,51,52,53,54,55,56,57,58,59,60,61,62,63,64,65,66,67,68,69,70,72,76,77,78,79,82,85,86,87,88,89,92,93,96,111,112,113,114,115,116,117,118,119,120,121,122,123,124,125,126,127,128].

	*C. albicans*	*C. glabrata*	*C. krusei*	*C. parapsilosis*	*C. tropicalis*
**Modification of drug target (protein or pathway)**	SNPs reducing the inactivation of *CaERG11* by azoles Overexpression of *CaERG11*	Not found	SNPs identified in *CkERG11* in resistant isolates Mild overexpression of *CkERG11*	Overexpression of *CpERG11*	SNPs reducing the inactivation of *CtERG11* by azoles Overexpression of *CtERG11*
	SNPs inactivating *CaERG3* or *CaERG5/CaERG11* to bypass the inactivation of ergosterol biosynthesis by azoles	SNPs inactivating *CaERG3* or *CaERG5/CaERG11* to bypass the inactivation of ergosterol biosynthesis by azoles Decreased expression of an acylCoA:sterol acyltransferase resulting in low sterol esterification	-	SNPs that inactivate *CpERG11* or *CpERG2* to bypass the inactivation of ergosterol biosynthesis by azoles	-
**Increased activity of drug-efflux pumps**	Overexpression of *CaCDR1*, *CaCDR2*, *CaMDR1*, *CaPDR16* Increased activity of CaTac1, CaMrr1, CaCap1, CaUpc2	Overexpression of *CgAQR1*, *CgCDR1*, *CgFLR2*, *CgPDH1, CgQDR2*, *CgSNQ2*, *CgTPO1*_1, *CgTPO1*_2, *CgTPO3* Increased activity of CaTac1, CaMrr1, CaCap1, CaUpc2	Overexpression of *CkABC1* and *CkABC2*	Overexpression of *CpMDR1* and *CpCDR1* Increased activity of CpUpc2	Overexpression of *CtCDR1* and *CtMDR1*

**Table 4:** Summary of the mechanisms of resistance to echinocandins, polyenes and 5-flucytosine (5-FC) reported in resistant *Candida* clinical isolates based on results from [50,92,93,94,95,96,99,100,101,102,103,105,106,107,108,109,110].

		*C. albicans*	*C. glabrata*	*C. krusei*	*C. parapsilosis*	*C. tropicalis*
**Modification of drug target/pathway**	**Echinocandins**	SNPs reducing the inactivation of *CaGSC1* by echinocandins	SNPs reducing the inactivation of *CgFKS1* and/or *CgFKS2* by echinocandins	SNPs reducing the inactivation of *CgFKS1* by echinocandins	SNPs reducing the inactivation of *CgFKS1* by echinocandins	SNPs reducing the inactivation of *CgFKS1* by echinocandins
	**Polyenes**	SNPs inactivating *ERG3* resulting in reduced ergosterol in the membrane	SNPs inactivating *ERG2*, *ERG6* or *ERG11* hypothesized to result in reduced ergosterol in the membrane	-	-	SNPs inactivating Ct*ERG11* hypothesized to result in reduced ergosterol in the membrane
	**5-Flucytosine**	SNPs reducing the inactivation of *CaFUR1* or *CaFCA1* by 5-FC Potential inactivation of *CaFCY21* or *CaFCY22*	SNPs reducing the inactivation of *CgFUR1* by 5-FC	-	SNPs reducing the inactivation of *CpFUR1* by 5-FC	Possible hyper activation of CtUra3 to increase formation of UMP

**Table 5:** Examples of reported complexes involving different metallic centers that were shown to exhibit interesting activity against *C. albicans.*

Metallic Center	Ligand	MIC/Diameter of Inhibition of the Complex (or Ligand) against C. albicans^*^	Ref
Ag	Dicarboxylic acid Phenanthroline	1–490 mM (ligand has antifungal activity at >1000 mM) 0.9–1.7 mM (ligand has antifungal activity at 149.4 mM)	[176]
	Phenanthroline	7.8 µg/mL (ligand has antifungal activity at 31.25 µg/mL)	[177]
	Tetrazole nitrogen	0.62–1.25 µg/mL (information regarding the activity of the ligand was not provided)	[148]
	Phenanthroline	12–113 mM (ligand has antifungal activity at 5000 mM)	[178]
	Benzimidazolydine	18 mm (ligand has no antifungal activity)	[146]
Cu	Schiff base	4 µg/mL (information regarding the activity of the ligand alone is not provided)	[179]
	Benzimidazolydine	12 mm (ligand has no antifungal activity)	[146]
	Azo dye	11 mm diameter (10 mm attributable to the ligand)	[152]
	Schiff base type	115 mM (ligand has antifungal activity at 245 mM)	[153]
	Schiff base + 2,2′-bipyridine ancillary	57 mM (ligand has antifungal activity at 188 mM)	[154]
	Chromone hydrazines	24.8 and 30.7 mm diameter (20.8 and 21.2 mm attributable to ligands)	[155]
	Dendrimer	1 mg/mL (ligand has antifungal activity at 12.9 mg/mL)	[156]
	Ferrocenyl chalcone derivatives	17 and 21 mm diameter (12 and 19 mm attributable to ligand)	[158]
	Tetradentate macrocyclic	22 mm diameter (16 mm attributable to ligand)	[157]
Co	Schiff base type ligand	32 µg/mL (information regarding the activity of the ligand alone is not provided)	[179]
	azo dye ligand	11 mm diameter (10 mm attributable to ligand)	[152]
	Schiff base type ligand	57–75% inhibition (40–60% attributable to ligand)	[147]
	Schiff base type ligand	125 mM (ligand has antifungal activity with 245 mM)	[153]
	Schiff base type ligand	82 mM (ligand has antifungal activity at 188 mM)	[154]
	Dendrimer ligand	0.6 mg/mL (ligand has antifungal activity at 12.9 mg/mL)	[156]
	Tetradentate macrocyclic ligand	22 mm diameter (15 mm attributable to ligand)	[157]
	Ethylenediamine derivatives	62.5 µg/mL (information regarding the activity of the ligand alone is not provided)	[180]
Ni	Bidentate azodye ligand	15.7 mm diameter (ligand has no antifungal activity)	[150]
	Schiff base type ligand	129 mM (ligand has antifungal activity with 245 mM)	[153]
	Schiff base type ligand + 2,2′-bipyridine ancillary ligand	87 mM (ligand has antifungal activity at 188 mM)	[154]
	Chromone hydrazone	22.5 and 25.6mm diameter (20.8 and 21.2 mm attributable to ligand)	[155]
	Dendrimer ligand	0.6 mg/mL (ligand has antifungal activity at 12.9 mg/mL)	[156]
	Tetradentate macrocyclic ligand	19 mm diameter (15 mm attributable to ligand)	[157]
Cd	Bidentate azodye ligand	17.1 mm diameter (ligand has no antifungal activity)	[150]
	Ferrocenyl chalcone derivatives	20 mm diameter (12 mm attributable to ligand)	[158]
Sn	Dithiocarbamate derivatives	2.5–250 µg/mL (information regarding the activity of the ligand alone is not provided)	[149]
	Schiff base type ligand	135 mM (ligand has antifungal activity with 245 mM)	[153]
	Schiff base type ligand + 2,2′-bipyridine ancillary ligand	102 mM (ligand has antifungal activity at 188 mM)	[154]
	Chromone hydrazine ligand	24.8 and 26.3 mm diameter (20.8 and 21.2 mm attributable to ligand)	[155]
Fe	Thiazole derivatives ligand	18.9 mm diameter (11.9 mm attributable to ligand)	[181]
	Ferrocenyl chalcone derivatives	17 mm diameter of inhibition zone (12 mm attributable to ligand)	[158]
	Bidentate azodye ligand	19.6 mm diameter (ligand has no antifungal activity)	[150]
	Ferrocenyl chalcone derivatives	15 mm diameter (12 mm attributable to ligand)	[158]
Ru	Perylene ligand	125 mM (information regarding the activity of the ligand alone is not provided)	[159]
Pb	Ferrocenyl chalcone derivatives	17 and 21 mm diameter (12 and 19 mm attributable to ligand)	[158]
Ba	Ferrocenyl chalcone derivatives	13 mm diameter (12 mm attributable to ligand)	[158]
Pd	Phenylphosphine ligand	0.5 µg/mL (information regarding the activity of the ligand alone is not provided)	[182]

**Table 6:** List of probiotics and *Candida* spp. by which these probiotics show antagonistic activity.

Probiotic	*Candida* spp.
*Lactobacillus rhamnosus* GG (ATCC 53103), *L. rhamnosus LC705*, *Propionibacterium freudenreichii* subsp. *shermanii* JS	*C. albicans*, *C. glabrata*, *C. krusei* and *C. tropicalis*
*Lactobacillus casei* and *Bifidobacterium breve*	*C. albicans*, *C. tropicalis*, *C. guillermondii*, *C. glabrata*, *C. krusei*, *C. kefyr* and *C. parapsilosis*
*L. rhamnosus* HS111, *L. acidophillus* HS101, and *Bifidobacterium bifidum*	*C. albicans*, *C. guillermondii,* *C. tropicalis*, *C. glabrata,* *C. dubliniensis*, *C. famata* and *C. parapsilosis*
*L. acidophilus, L. rhamnosus*, *L. delbrueckii* subsp. *bulgaricus* and *S. thermophiles*	*Candida* spp.
*L. rhamnosus* GR-1 and *L. reuteri* RC-14	*C. albicans* and *non-C. albicans*
*Lactobacillus fermentum* LF10 and *L. acidophilus* LA02	*C. albicans*, *C. glabrata*, *C. parapsilosis* and *C. krusei*
*Bifidobacterium* and *Lactobacillus (DanActive or yoPlus yogurt)*	*C. albicans* and *non-C. albicans*
*L. casei* subsp. *rhamnosus*	*C. albicans* and *non-C. albicans*
*L. reuterii* ATCC 55730 and *L. rhamnosus* (ATCC 53103)	*Candida* spp.
*L. acidophillus*, *L. rhamnosus*, *B. longum*, *B. bifidum*, *S. boulardii*, and *Saccharomyces thermophiles*	*Candida* spp.
*L. acidophilus*, *Bifidobacterium lactis*, *B. longum*, and *B. bifidum*	*C. albicans* and *C. glabrata*
